# Aberrant hepatic arteries running through pancreatic parenchyma encountered during pancreatoduodenectomy: Two rare case reports and strategies for surgical treatment

**DOI:** 10.1097/MD.0000000000003867

**Published:** 2016-12-09

**Authors:** Lei Wang, Jianwei Xu, Dong Sun, Zongli Zhang

**Affiliations:** Department of General Surgery, Qilu Hospital, Shandong University, Jinan, Shandong Province, China.

**Keywords:** aberrant artery, hepatic artery, pancreatoduodenectomy

## Abstract

**Background:**

Aberrant hepatic arteries (HAs) encountered during pancreatoduodenectomy are difficult to manage.

**Mehtods:**

Two cases with rare types of aberrant HA running through the pancreatic parenchyma were reviewed.

**Results:**

The first case, a 68-year-old man, was admitted with obstructive jaundice. A tumor of the pancreatic head and aberrant HAs were suspected on computed tomography (CT) scan. At laparotomy, a new variation was identified; namely, 2 aberrant arteries—a right replaced HA and middle HA (RMHA) that both originated from the superior mesenteric artery (SMA) and ran via intrapancreatic paths posterior and anterior to the pancreatic head, respectively. Branches of the RMHA to the pancreas were ligated and severed and the trunk preserved. The RMHA was mistakenly identified as an aberrant left HA (RLHA), whereas the RLHA was overlooked and not dissected intraoperatively. CT angiography performed 11 days postoperatively identified that the RLHA originated from the left gastric artery (LGA).

The second case had a variation of Michels IX. A 58-year-old woman presented with obstructive jaundice and a distal cholangiocarcinoma was suspected on the basis of enhanced CT scan. At laparotomy, the common hepatic artery (CHA) was found to originate entirely from the SMA and run posterior to the pancreatic head via an intrapancreatic path. The segment of CHA in the pancreatic parenchyma was removed and reconstructed with the LGA.

**Conclusions:**

Preoperative identification of aberrant HAs helps in planning appropriate operative procedures and minimizing unnecessary complications. Both preservation and reconstruction of these arteries are technically safe and feasible; however, preservation is preferable.

## Abstract 2

Supplemental Digital Content is available in the text

## 1 Introduction

Anatomical abnormalities of hepatic arteries (HAs) are common, only 52% to 80% of patients showing “normal anatomy”; that is, the common hepatic artery (CHA) originating from the celiac trunk.^[1]^ Based on the anatomy of 200 cadaver livers, Michels described the 10 most common variants of arterial blood supply to the liver (Table 1).^[2]^ Hiatt et al^[3]^ simplified Michel classification by proposing a classification scheme with 6 arterial variants. Although not all vascular abnormalities of the hepatic artery (HA) affect the course or outcomes of pancreatoduodenectomy (PD),^[4]^ identification of aberrant HAs is important when performing this procedure because such variations can necessitate altering the surgical approach and interfere with resection and reconstruction of the digestive tract.^[4,5]^

**Table 1:**
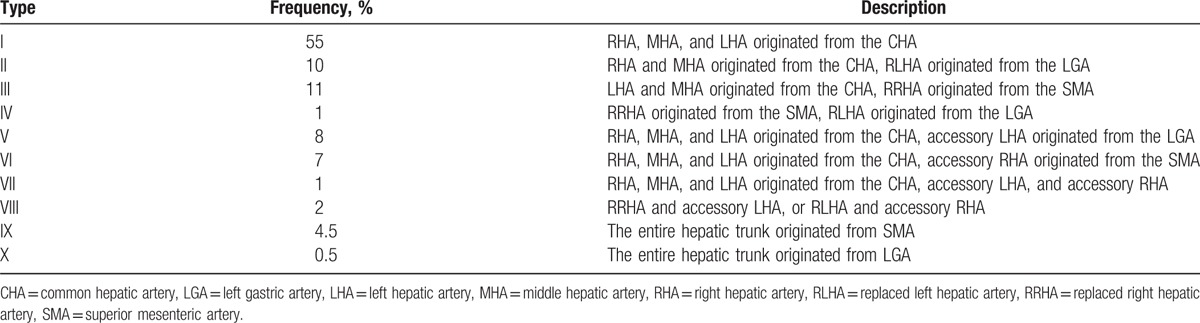
Types and frequency of aberrant hepatic arteries according to the Michel classification.

In the current report, we present 2 cases of aberrant HAs running through the pancreatic parenchyma encountered during PD and the surgical strategies that were used to manage them. In one case, whose type of aberrant artery is not covered by Michel classification, the aberrant arteries were preserved during PD. The other case had a version of Michels IX and underwent arterial reconstruction.

## 2 Case 1

**Figure 1:**
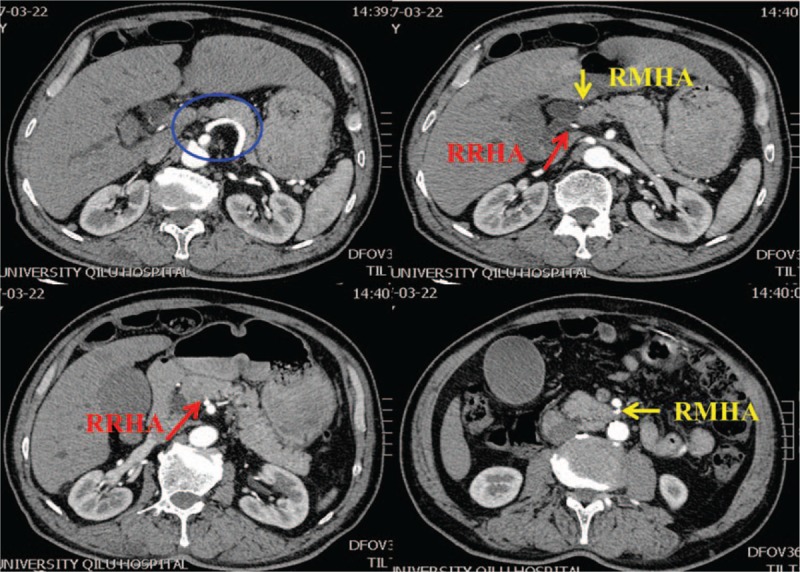
Case 1: preoperative computed tomography scan images showing aberrant hepatic arteries. A right and middle hepatic artery are arising from the superior mesenteric artery (SMA).

This patient's aberrant artery was of a type not covered by Michel classification. A 68-year-old man was admitted to our department with obstructive jaundice for the previous 15 days. Relevant laboratory findings included serum alanine transferase (148 U/L), gamma-glutamyl transferase (1287 U/L), total bilirubin (259.7 μmol/L), and CA-199 (26.2 U/mL). A tumor of the pancreatic head and uncinate process was suspected on the basis of dynamic computed tomography (CT) findings. Additionally, aberrant HAs were observed, comprising both a right hepatic artery (RHA), that is, a replaced RHA (RRHA) and a replaced middle hepatic artery (RMHA), both of which arose from the superior mesenteric artery (SMA) (Fig. 1, see Figure, Supplemental Content 1, which illustrates the running path of MHA in liver). The left hepatic artery (LHA) originated from the left gastric artery (LGA); that is, it was a replaced LHA (RLHA) (see Figure, Supplemental Content 2, which illustrates the origination of RLHA). This type of aberrant HA was not described by Michels,^[2]^ nor since to our knowledge. However, some aspects of these aberrant HAs were not accurately identified when the CT images were read preoperatively. The MHA was mistakenly identified as the RLHA, whereas the actual RLHA was overlooked. The details described in this report were identified at laparotomy and by CT angiography (CTA) postoperatively.

**Figure 2:**
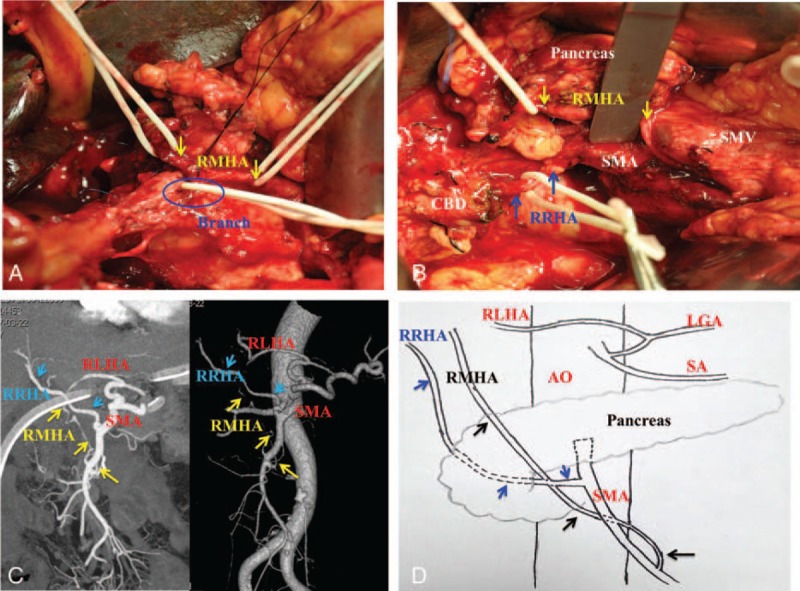
Case 1: details of aberrant hepatic arteries observed during laparotomy and by postoperative CT angiography. (A) Dissection of the RMHA revealed that it was running anterior to the head of pancreas and taking an intrapancreatic path before irrigating the liver (yellow arrows). Branches of the RMHA to the pancreas were ligated (blue). (B) The replaced right and middle hepatic arteries (RRHA and RMHA) were arising from the SMA (RRHA: blue arrows; RMHA, yellow arrows). (C) Postoperative CTA image showing the aberrant hepatic arteries. (D) Schematic diagrams of the aberrant hepatic arteries. CT = computed tomography, CTA = CT angiography, LGA = left gastric artery, RLHA = replaced left hepatic artery, RMHA = replaced middle hepatic artery, RRHA = replaced right hepatic artery, SMA = superior mesenteric artery.

PD was performed. At laparotomy, an RHA (i.e., RRHA) originating from the SMA and running posterior to the head of pancreas was identified and dissected from the pancreas (Fig. 2). The MHA (i.e., RMHA) was running anterior to the head of pancreas and taking an intrapancreatic path before supplying the liver. There was no definite evidence of arterial invasion. Branches of the MHA to the pancreas and duodenum were ligated and severed and the trunk preserved (Fig. 2). Unfortunately, the MHA was mistakenly identified as the RLHA, whereas the LHA was overlooked and not dissected. Pathologic examination of the operative specimen showed a well to moderately differentiated pancreatic ductal adenocarcinoma of stage T3N0M0 based on the National Comprehensive Cancer Network guidelines (Version 2. 2015). Histopathologically, an R0 resection had been achieved. CTA performed 11 days postoperatively identified the RLHA (Fig. 2). Eleven days after surgery, a pancreatic fistula was diagnosed and continuous irrigation via drainage tube initiated. An intra-abdominal hemorrhage occurred 18 days postoperatively. Angiography showed a pseudoaneurysm rupture of the mesojejunal vessels of the pancreaticojejunostomy; this was treated by embolization. The patient was discharged 32 days postoperatively.

## 3 Case 2

**Figure 3:**
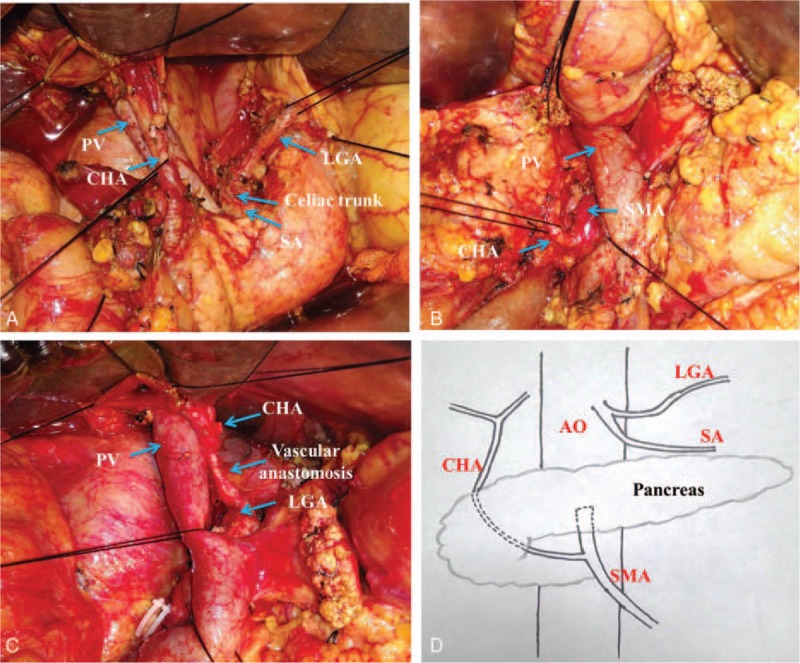
Case 2: details of aberrant hepatic artery observed during laparotomy. (A) An aberrant CHA was observed. (B) The CHA arose from the SMA and ran posterior to the head of pancreas. (C) The segment of CHA in the pancreatic parenchyma was removed and reconstructed with the LGA. (D) Schematic diagrams of the aberrant hepatic arteries. CHA = common hepatic artery, LGA = left gastric artery, PV = portal vein, SA = splenic artery, SMA = superior mesenteric artery.

This patient had a variation of Michels IX.^[2]^ A 58-year-old woman presented with obstructive jaundice and distal cholangiocarcinoma was suspected on the basis of enhanced CT images. In addition, CT images showed that there was no CHA arising from the celiac trunk. PD was performed. At laparotomy, the CHA was found to originate entirely from the SMA (Michels IX) and run posterior to the head of pancreas via an intrapancreatic path. The segment of CHA in the pancreatic parenchyma was removed and reconstructed with the LGA (Fig. 3). Pathologic examination of the surgical specimen showed a moderately differentiated distal cholangiocarcinoma of stage T3N0M0. CHA flow volume measured by ultrasonography 1 month after operation was 48 cm/s (normal 46–66 cm/s). The postoperative course was uneventful with no hepatic failure, abscess formation, or bile leakage.

The present study was approved by the Ethics Committee of Qilu Hospital, Shandong University. Both cases were collected and reported in accordance with approved guidelines of Shandong University. Written informed consent was obtained from all patients.

## 4 Discussion

Variations in the arterial blood supply to the liver are not uncommon, having an incidence of 24.3% according to Hiatt et al.^[3]^ Some variations can affect the course of PD, especially if encountered intraoperatively rather than being identified during planning of the procedure, and increase the rate of surgical complications, such as pancreatic fistula, bleeding, hepatic ischemia or failure, and bile leakage.^[6]^ Awareness of arterial aberrance and modifying surgical strategies accordingly may prevent unnecessary morbidity and mortality. We here report not only a previously undescribed form of aberrant HA but also describe 2 surgical strategies for dealing with aberrant HAs running through the pancreatic parenchyma and encountered during PD.

When PD is scheduled, identifying any aberrant blood supply to the liver is essential, enabling appropriate planning of the procedure and minimization of complications. Failing to recognize aberrant HAs preoperatively may necessitate abandoning radical resection, as was performed in one of our previous cases. This female patient at the age of 56 years old presented with jaundice and ampullary carcinoma was suspected on CT scan. An aberrant CHA was discovered intraoperatively running posterior to the pancreatic head and originating from the SMA. Lacking the required planning and expertise, the surgeon elected to perform a choledochojejunostomy rather than a PD. Thus, correct interpretation of CT scan findings preoperatively is important,^[6,7]^ especially focusing on the running path of hepatic arteries. If abnormalities of celiac trunk, or abnormal branches originated from SMA are observed, aberrant hepatic arteries should be highly suspected. Although angiography can provide more details about aberrant arteries, it does not identify the relationship between the paths of the arteries and the pancreatic parenchyma, which is important information when attempting to optimize presurgical planning. Angiography is advocated only when very complex or rare arterial anomalies have been found by CT or magnetic resonance imaging scans.^[6,8]^ Although some details were not consistent with those identified during laparotomy, both the exceedingly uncommon forms of aberrant HAs in the current 2 cases were suspected preoperatively based on enhanced CT scan images. We now recommend preoperative CTA in patients with periampullary carcinoma who are suspected of having aberrant HAs to evaluate resectability and degree of vascular involvement and facilitate planning of surgical strategies. Although palpating the tumor–vessel relationship and the path of an artery is useful for characterizing aberrant arteries, even experienced surgeons can find this difficult. Identifying the optimal surgical approach preoperatively would facilitate management of aberrant arteries and minimize accidental injury or ligation. For example, a ventral approach to dissecting an RRHA or an early retropancreatic dissection to expose the SMA may be useful.^[9,10]^

Accidental injury or ligation of hepatic arteries may result in intraoperative bleeding and hepatobiliary ischemia, which is always associated with severe and costly morbidities.^[11]^ Additionally, failure to preserve the arterial blood supply to the liver is particularly serious in patients undergoing PD because biliary ischemia may lead to biliary leakage or an anastomotic ischemic stricture.^[12]^ As we have described in the current cases, there are 2 possible strategies for managing aberrant HAs; namely, preserving the trunk of the aberrant artery or removing it and performing arterial reconstruction. Both procedures are technically safe and feasible.

Dissection and preservation of aberrant HAs may be performed as the first choice. The potential risks of this procedure are increased intraoperative blood loss and incidence of positive margins, which may also result in pancreatic fistula. Additionally, overzealous dissection may result in injury to the artery, which is a risk factor for postoperative hemorrhage. However, pathologic examination reportedly did not show an increased incidence of positive margins in 1 series of patients whose accessory RHAs were preserved or replaced.^[13]^ An R0 resection was achieved in the first case in our study. Although postoperative hemorrhage did occur, the bleeding vessel was not one of the aberrant HAs, making it difficult to be sure whether there was an association between the postoperative hemorrhage and preservation of the aberrant HAs; it was possibly a coincidence. However, preservation of aberrant HAs is not always possible. An en bloc resection, transaction, and reconstruction may be performed by primary anastomosis or through implantation at a suitable arterial site in patients whose arteries are invaded or encased by their tumors.^[14]^ In any case, dealing with aberrant HAs is technically challenging for most surgeons. We performed arterial reconstruction in our second case, not because of arterial invasion by tumor, but because of failure to identify the CHA arising from the SMA and the consequent inadequacy of preoperative planning, including a large amount of blood transfusion preparation, and lack of understanding the feasibility and risk of artery preservation.

Several other strategies are possible, including sacrifice and preoperative embolization. Sacrifice is appropriate for accessory RHAs of small caliber that are impeding tumor resection and do not present clinically significant results if resected.^[15]^ Although preoperative embolization can decrease liver blood flow, it is not routinely recommended because of the high incidence of complications.^[13,16]^ Additionally, neoadjuvant therapy is an alternative in selected patients with aberrant HA invasion or encasement.^[13]^

In conclusion, variations in the arterial blood supply to the liver are common. Knowledge of these abnormalities preoperatively helps with planning of an appropriate procedure and minimization of complications. Adopting individualized surgical strategies may prevent unnecessary morbidity and mortality. Both preservation and arterial reconstruction are technically safe and feasible, the former being the treatment of choice when possible.

## Acknowledgment

The authors are grateful to Jingdu Yan for drawing schematic diagrams of the anatomy.

## Supplementary Material

Supplemental Digital Content
