# Supplementary material for: Aberrant hepatic arteries running through pancreatic parenchyma encountered during pancreatoduodenectomy: Two rare case reports and strategies for surgical treatment
Source: Medicine (Baltimore). 2016 Dec 9;95(49):e3867. doi: 10.1097/MD.0000000000003867 (PMC5265976; doi:10.1097/MD.0000000000003867)
Supplement: Supplemental Digital Content [file medi-95-e3867-s001.doc]

**Supplemental Digital Content 1.** Figure that illustrates the running path of MHA in liver (the red circle).

**Supplemental Digital Content 2.** Figure that illustrates the origination of RLHA**.** RLHA, replaced left hepatic artery
